# Low-Dose Vitamin D3 Supplementation: Associations with Vertebral Fragility and Pedicle Screw Loosening

**DOI:** 10.3390/jcm14228052

**Published:** 2025-11-13

**Authors:** Jun Li, André Strahl, Beate Kunze, Stefan Krebs, Martin Stangenberg, Lennart Viezens, Patrick Strube, Marc Dreimann

**Affiliations:** 1 Spine Center for Neuroorthopaedics, Spinal Cord Injuries, and Scoliosis, RKH Orthopedic Clinic Markgröningen, 71706 Markgröningen, Germany; 2 Department of Psychosomatic Medicine and Psychotherapy, Centre for Internal Medicine, University Medical Center Hamburg-Eppendorf, 20246 Hamburg, Germany; 3 Department of Spinal Surgery and Neurosurgery, Tabea Hospital Hamburg, 22587 Hamburg, Germany; 4 Department of Trauma and Orthopaedic Surgery, Division of Spine Surgery, University Medical Center Hamburg-Eppendorf, 20246 Hamburg, Germany; 5 Department of Orthopaedics, Jena University Hospital, Campus Waldkliniken Eisenberg, 07607 Eisenberg, Germany

**Keywords:** vertebral fragility, pedicle screw loosening, ageing population, vitamin D3 supplementation, posterior instrumented spinal fusion, spine surgery

## Abstract

**Background/Objectives**: Vitamin D deficiency contributes to pathological vertebral fragility (path-VF), including fragility fractures and early pedicle screw loosening after posterior instrumented spinal fusion (PISF). Supplementation practices remain inconsistent. This retrospective study evaluated whether patients with path-VF receive appropriate vitamin D3 (Vit.D3) supplementation and assessed the dose–response relationship between daily intake and path-VF risk, particularly in older adults. **Methods**: A total of 210 patients treated with kyphoplasty or PISF (2022–2023) were classified into a path-VF or control group. Daily oral Vit.D3 intake was categorised as Zero- (0 IU), Low- (<2000 IU), or High-Dose (≥2000 IU). Statistical analyses were performed for each dosage group, including subgroup analyses for patients aged ≥67.5 years. Vertebral BMD was estimated using mean Hounsfield Units (HU) from T11–L5. **Results**: Patients in the path-VF group received significantly lower Vit.D3 doses than controls (1431.4 ± 1055.7 vs. 2366.7 ± 1186.7 IU/day, *p* < 0.001). Low-dose supplementation was associated with a markedly increased risk of path-VF compared with high-dose in the overall cohort (OR = 6.5, *p* = 0.003) and in patients aged ≥67.5 years (OR = 8.6, *p* = 0.008). Logistic regression identified a threshold of 1900 IU/day (AUC = 0.805). Mean vertebral HU values were significantly lower in the path-VF group than in controls (71.9 ± 29.1 vs. 133.5 ± 52.6, *p* < 0.001), and no consistent HU gains were observed with increasing Vit.D3 dosage. **Conclusions**: Low-dose Vit.D3 supplementation was associated with increased path-VF risk, especially in patients aged >67.5 years. Patients without path-VF had received significantly higher doses, suggesting broader benefits of adequate Vit.D3 beyond bone density. A daily intake above 1900 IU may serve as a practical threshold for at-risk elderly patients.

## 1. Introduction

Vitamin D (Vit.D) deficiency is a well-recognised risk factor for impaired spinal health and is closely associated with pathological vertebral fragility (path-VF). It increases the risk of fragility fractures (FF) [1], delays postoperative recovery and physical rehabilitation [2], reduces spinal fusion success rates [3], and contributes to implant-related complications such as early pedicle screw loosening (PSL). Despite its clinical relevance, the risks of path-VF are often insufficiently assessed in clinical practice prior to posterior instrumented spinal fusion (PISF). Only 44% of spine surgeons perform preoperative bone mineral density (BMD) evaluations, and just 12% assess metabolic laboratory parameters, including 25-hydroxyvitamin-D (25(OH)D), parathyroid hormone, and calcium levels [4].

Although the above considerations support the role of Vit.D, perioperative supplementation in bone surgeries, especially in spine surgery, has shown inconsistent results. Some study reported improved fusion rates, symptom relief, and recovery despite small sample sizes [3]. Others, show no clear postoperative benefits and no consistent dose–response relationship [5,6]. Similar inconsistencies are seen in broader orthopedic research, often limited by heterogeneous designs and low methodological quality [7,8].

The biomechanical integrity of the vertebrae is maintained through dynamic bone remodelling, adapting to internal and external influences such as ageing, nutrition, biomechanical stress, menopause, chronic inflammatory conditions, and other systemic disorders [9,10]. Disruption of this osteogenic–osteoclastic balance significantly reduces BMD, predisposing vertebrae to fragility and increasing the risk of path-VF [11]. Vit.D is crucial for bone development and mechanical strength, facilitating calcium and phosphate metabolism [12,13]. Among available Vit.D supplements, vitamin D3 (Vit.D3) demonstrates superior efficacy in elevating serum 25(OH)D concentrations compared with vitamin D2 (Vit.D2) [14].

Existing international guidelines for Vit.D3 supplementation in adults vary widely in both age grouping and dosage and often provide ambiguous recommendations regarding target populations (typically limited to categories such as infants, children, adults, and pregnant or lactating women), initiation timing, and optimal dosage. Some countries make no age distinction for adults, while others introduce multiple age categories but recommend almost identical adequate intake levels [15,16,17,18,19]. Internationally [15,16,17,18,19], daily doses range from as low as 9 μg (360 IU) to as high as 100 μg (4000 IU). This discrepancy fosters subjective decision-making in clinical practice, frequently overlooking certain patient groups (particularly males) and leading to arbitrary dosing within this broad range.

This study was performed to evaluate Vit.D3 supplementation in spinal surgery patients and to determine whether those at risk of path-VF (including FF and early PSL following PISF) receive appropriate supplementation compared with those without such risk. In addition, this study sought to identify specific cut-off values for age and daily Vit.D3 dosage that may guide supplementation strategies. The findings may provide evidence to refine clinical guidelines for Vit.D3 supplementation, with the aim of reducing the incidence of path-VF and its associated complications in spinal surgery patients.

## 2. Materials and Methods

### 2.1. Patients

In this retrospective case–control study, we evaluated 210 patients who underwent spinal surgical treatments, including balloon kyphoplasty and PISF, in the thoracic and lumbar regions at a single spine centre between 2022 and 2023.

### 2.2. Definitions of Path-VF and Early PSL

For this study, the diagnostic criteria for path-VF were based on the framework established in a previous study [20], with adaptations to the present research context. A diagnosis of path-VF was considered if any of the following conditions were met, after histological exclusion of tumour-related fractures:

(1) cases requiring balloon kyphoplasty for path-VF,
(2) intraoperative use of cement-augmented pedicle screws during the first PISF, or
(3) occurrence of non-traumatic PSL within 6 months after the initial PISF in the absence of prior screw reinforcement.

The indications for PISF in this study included severe degenerative lumbar stenosis with instability, idiopathic scoliosis, advanced spondylolisthesis, and de novo lumbar scoliosis. Patients with spondylodiscitis or spinal tumours were excluded.

Among the available techniques for enhancing screw fixation in osteoporotic vertebrae, cement augmentation is regarded as the most widely used and effective [21,22]. Therefore, only cement-augmented screws were used in the present cohort, while other reinforcement options (e.g., expandable screws, specialised thread designs, or bioactive-coated implants) were excluded.

The decision to perform screw reinforcement was made by senior spine surgeons following thorough preoperative assessment. Consistent with earlier findings, early PSL (<6 months) is predominantly associated with low BMD and reduced bone quality [23], whereas later PSL (>1 year) is more often related to mechanical overload, adjacent segment degeneration, suboptimal implant positioning, or chronic low-grade infection [24,25,26]. Based on these considerations, a 6-month threshold was applied in this study to define early PSL.

PSL was diagnosed using computed tomography (CT) imaging based on either a radiolucent rim > 1 mm around the screw or signs of screw pull-out/cut-out [26]. To reduce radiation exposure and costs, CT was limited to patients with severe symptoms such as intense localised pain or significant activity restriction [27].

### 2.3. Grouping

Patients were classified into two cohorts: the path-VF group, comprising individuals diagnosed with path-VF, and the control group, comprising patients without evidence of path-VF. Preoperative demographic data, including age, sex, and daily Vit.D3 supplementation dosage, were recorded for all participants.

### 2.4. Categorisation of Vit.D3 Dosage

Data on vitamin D supplementation were retrieved from general practitioner medication records or, when unavailable, collected during preoperative patient interviews.

To assess the impact of daily Vit.D3 intake, patients were categorised into three dosage groups: Zero-Dose (0 IU/day), Low-Dose (<2000 IU/day), and High-Dose (≥2000 IU/day). Patients whose Vit.D3 supplementation dosage changed between the PISF and the 6-month follow-up were excluded from the analysis.

Based on our previous study [20], which identified 67.5 years as the optimal age threshold for predicting path-VF risk, analyses were performed for the overall cohort and further stratified into subgroups above and below this threshold to examine the relationship between Vit.D3 intake and path-VF risk.

### 2.5. Measurement of Vertebral Hounsfield Units (HU)

To validate the definition of path-VF used in this study and to confirm the robustness of the subsequent grouping, vertebral bone quality was assessed using preoperative CT-based HU measurements. Among the 210 patients, only 8 had dual-energy X-ray absorptiometry (DEXA) results available before surgery. Preoperative lumbar spine CT scans were retrospectively evaluated in 88 patients. HU values were measured from Th11 to L5 using elliptical region of interest placed in the cancellous bone between the upper and middle thirds of each vertebral body, excluding cortical areas. Regions containing vascular structures, Schmorl’s nodes, sclerosis, or tumorous changes were excluded. For each patient, the mean HU value from Th11 to L5 was calculated and used in subsequent analyses.

### 2.6. Statistical Analysis

All statistical analyses were conducted using R software version 4.4.1, 14 June 2024 (The R Foundation for Statistical Computing, Vienna, Austria). Continuous variables are reported as mean ± standard deviation, and statistical significance was defined as a two-tailed *p*-value of <0.05.

To compare means of numeric variables, the data normality was assessed previously using the Shapiro–Wilk test and Kolmogorov–Smirnov test, and variance homogeneity was verified using Levene’s test and the F-test. Due to non-normality, variance inhomogeneity, or limited sample size, group comparisons for age, daily Vit.D3 dosage, and HU values were performed using the Mann–Whitney U test. Effect sizes were reported as rank-biserial correlation coefficients (*r*), with *r* > 0.3 and *r* > 0.5 interpreted as medium and large effects, respectively.

Associations between Vit.D3 dosage categories and the risk of path-VF or path-VF with early PSL were examined using Fisher’s exact test, yielding odds ratios (ORs) with corresponding 95% confidence intervals (CIs).

To further explore potential predictors, multiple logistic regression was performed, incorporating sex, age, and Vit.D3 dosage categories (Zero-, Low-, and High-Dose) as independent variables. Multicollinearity among predictors was evaluated using variance inflation factors (VIF). A VIF < 5 was considered acceptable. Receiver operating characteristic (ROC) curve analysis was used to identify potential dosage thresholds, with an area under the curve (AUC) of ≥0.8 interpreted as indicating excellent discriminative ability.

## 3. Results

### 3.1. Demographics of the Study Cohort

The study cohort comprised 210 patients with a mean age of 67.5 years (range 18.5–87.9 years), including 80 males and 130 females. Of these, 84 patients were classified into the path-VF group and 126 into the control group. Osteoporosis was pre-diagnosed in 42 patients using DEXA or quantitative CT, of whom 13 did not take Vit.D3, 20 received a low dose, and 9 received a high dose. In total, 53 patients in the cohort received Vit.D3 supplementation at varying doses. Detailed demographic characteristics are summarised in Table 1.

### 3.2. HU Values in the Path-VF and Control Groups

As expected, HU measurements confirmed the validity of the applied path-VF definition and the derived grouping. The path-VF group showed significantly lower mean vertebral HU values than did the control group (71.9 ± 29.1 vs. 133.5 ± 52.6, *p* < 0.001, *r* = 0.61), consistent with reduced bone quality in patients meeting the path-VF criteria.

### 3.3. Differences in Daily Doses of Vit.D3 Between the Path-VF and Control Groups

Oral daily Vit.D3 supplementation doses were compared between the path-VF and control groups using the Mann–Whitney U test. Patients in the path-VF group received significantly lower daily doses than those in the control group (Table 2).

**Figure 1:**
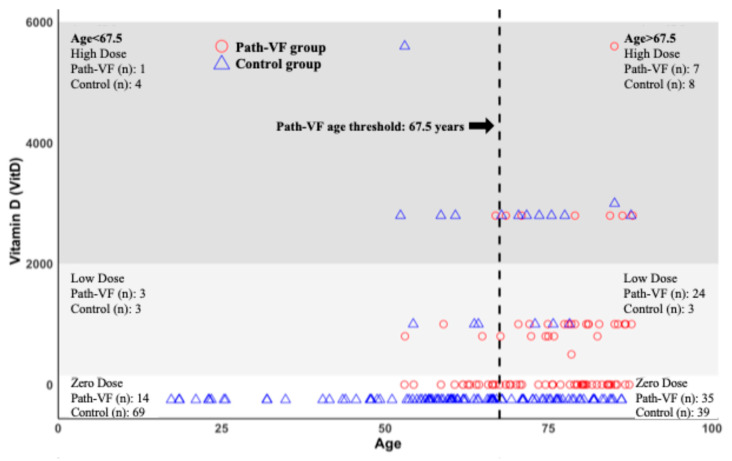
Distribution of daily vitamin D3 supplementation by age group and dosage category. Each dot represents an individual patient, with red circles indicating patients in the path-VF group and blue triangles indicating controls. The vertical dashed line marks the path-VF age threshold at 67.5 years. Vitamin D_3_ supplementation is categorized into Zero Dose (0 IU/day), Low Dose (<2000 IU/day), and High Dose (≥2000 IU/day). Numbers of patients per subgroup are indicated for both path-VF and control groups.

Figure 1 illustrates the age distribution of Vit.D3 supplementation. Patients without Vit.D3 supplementation and without path-VF risk were spread across all age ranges. By contrast, those not receiving Vit.D3 but meeting the path-VF criteria for FF or early PSL were mainly observed from approximately 50 years of age onwards. The previously established age threshold for path-VF risk (67.5 years) [20] is indicated by a vertical dashed line. Among patients receiving Vit.D3, daily doses below 2000 IU were predominantly found in the path-VF group, particularly in individuals above the 67.5-year threshold. By comparison, only 6 of 33 patients in the control group fell into this low-dose category. Conversely, most patients receiving ≥ 2000 IU/day (12 of 20) were in the control group and showed no path-VF risk.

### 3.4. ORs of Oral Vit.D3 Dosages for Path-VF Risk

**Figure 2:**
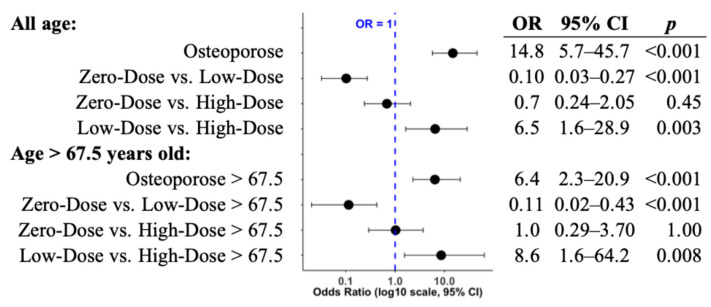
Forest plot of odds ratios (ORs) for path-VF risk across Vit.D3 dosage groups. ORs with 95% confidence intervals and *p*-values are shown for comparisons among Zero-, Low-, and High-Dose vitamin D_3_ groups in the overall cohort and in patients aged over 67.5 years. ORs are displayed on a log_10_ scale.

ORs for different oral Vit.D3 dosage categories and path-VF risk were calculated for the overall cohort (*n* = 210) and for the subgroup of patients aged >67.5 years (Figure 2), with diagnosed osteoporosis serving as a positive control.

As expected, osteoporosis was significantly associated with increased path-VF risk in both the overall cohort (OR: 14.8, 95% CI [5.7–45.7], *p* < 0.001) and the older subgroup (OR: 6.4, 95% CI [2.3–20.9], *p* < 0.001). The Vit.D3 dosage showed similar patterns in both analyses. Compared with patients taking Low-Dose Vit.D3, those in the Zero-Dose category had a significantly lower path-VF risk in the overall cohort (OR: 0.10, 95% CI [0.03–0.27], *p* < 0.001) and in the older subgroup (OR: 0.11, 95% CI [0.02–0.43], *p* < 0.001). This apparent lower risk in the Zero-Dose group than in the Low-Dose group will be further considered in the Discussion. Low-Dose supplementation was associated with a significantly higher path-VF risk than was High-Dose supplementation (overall OR: 6.5, 95% CI [1.6–28.9], *p* = 0.003; older subgroup OR: 8.6, 95% CI [1.6–64.2], *p* = 0.008). Zero-Dose versus High-Dose showed no significant association with path-VF (OR not different from 1; both *p*-values > 0.05).

**Figure 3:**
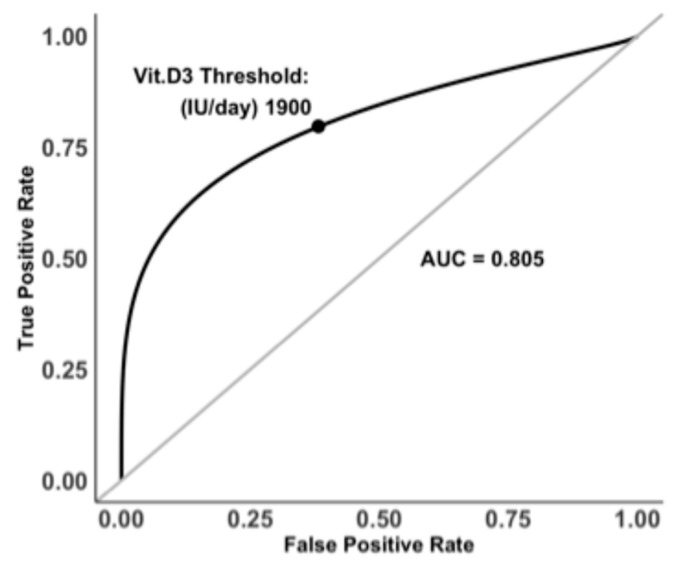
ROC curve identifying 1900 IU/day as the threshold of daily Vit.D3 intake for predicting path-VF (AUC = 0.805).

Multiple logistic regression analysis assessed the associations between path-VF occurrence and age, sex, and daily Vit.D3 dosage (Zero-, Low-, and High-Dose). All predictors showed VIF values close to 1 (range: 1.00–1.07), indicating no evidence of multicollinearity. As shown in Table 3, female sex was positively but not significantly associated with path-VF risk (*β* = 0.64, *p* > 0.05). Higher daily Vit.D3 dosage was linked to a small yet statistically significant reduction in path-VF occurrence (*β* = −0.001, *p* = 0.018). Increasing age was significantly associated with elevated path-VF risk (*β* = 0.08, *p* = 0.031). ROC analysis for daily Vit.D3 dosage identified a threshold of 1900 IU/day, yielding an AUC of 0.805 (Figure 3), indicating good predictive accuracy.

### 3.5. Effects of Different Vit.D3 Daily Doses on HU Values

**Figure 4:**
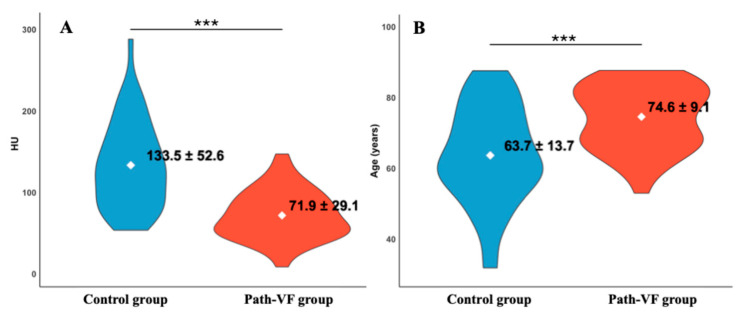
Violin plots comparing (**A**) vertebral HU values and (**B**) patient age between the control and path-VF groups. The path-VF group exhibited significantly lower HU and higher age (*** *p* < 0.001).

Firstly, HU values in the path-VF group were significantly lower than in the control group (*p* < 0.001) (Figure 4A). The violin plots show that HU values for path-VF patients were concentrated below 100, whereas control patients displayed a broader distribution towards higher values. By contrast, patient age showed the opposite pattern (Figure 4B), with those in the path-VF group being older (74.6 ± 9.1 years) than those in the control group (63.7 ± 13.7 years, *p* < 0.001, *r* = 0.42).

**Figure 5:**
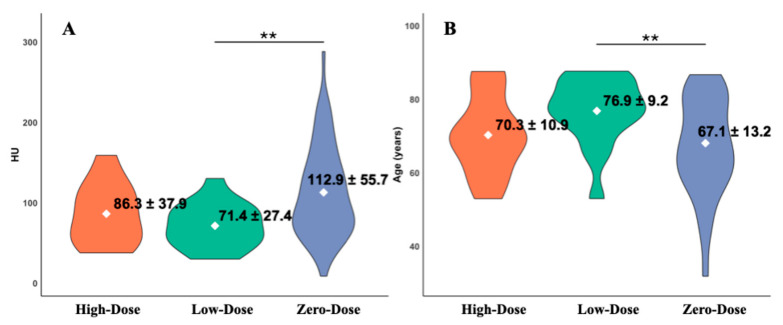
Violin plots comparing (**A**) vertebral HU values and (**B**) age among the High-, Low-, and Zero-Dose Vit.D_3_ groups. The Low-Dose group showed significantly lower HU and higher age than the Zero-Dose group (** *p* < 0.01), while comparisons involving the High-Dose group did not reach statistical significance.

We next examined the relationship between Vit.D3 dosage, HU values, and age. Mean HU values (Figure 5A) were 86.3 ± 37.9 (High-Dose, *n* = 11), 71.4 ± 27.4 (Low-Dose, *n* = 16), and 112.9 ± 55.7 (Zero-Dose, *n* = 61), with a statistically significant difference observed only between the Low-Dose and Zero-Dose groups (*p* = 0.004, *r* = 0.55). Corresponding mean ages (Figure 5B) were 70.3 ± 10.9, 76.9 ± 9.2, and 67.1 ± 13.2 years, respectively, with a significant difference again between the Low-Dose and Zero-Dose groups (*p* = 0.006, *r* = 0.53). All comparisons involving the High-Dose group showed no statistically significant differences, with small effect sizes (*r* < 0.3), suggesting that the limited sample size may have contributed to the lack of significance.

## 4. Discussion

Vit.D is a fat-soluble vitamin that plays a central role in regulating calcium, magnesium, and phosphorus metabolism. Serum 25(OH)D measurement is the standard method for assessing Vit.D status, with concentrations above 30 ng/mL generally regarded as sufficient [28]. Ageing, menopause, and reduced outdoor activity further exacerbate chronic Vit.D deficiency [29], increasing the risk of osteoporosis, path-VF with associated FF, and postoperative complications following instrumented spinal fusion [3].

### 4.1. Clinical Relevance in Spinal Surgery

In spinal surgery, Vit.D inadequacy is defined as serum 25(OH)D levels of 20–30 ng/mL, and deficiency as levels of <20 ng/mL [30]. While approximately 30% of the general population have levels below 20 ng/mL [31], the prevalence is markedly higher among patients requiring surgical treatment, with up to 73.6% for levels below 30 ng/mL and 36.8% for levels below 20 ng/mL [32]. Deficiency is linked to worse preoperative symptom severity, reflected in higher Japanese Orthopaedic Association, visual analogue scale, and Oswestry Disability Index (ODI) scores [28,33,34]. It is also associated with poorer postoperative recovery [2,35], longer fusion times [36], and lower fusion success rates [3].

Perioperative Vit.D3 supplementation has shown potential benefits in symptom relief and functional recovery, yet existing studies remain inconsistent regarding optimal dosage, timing, and patient selection [8]. Ko et al. used intramuscular injections of 100,000 IU perioperatively without specifying frequency [35], while Xu et al. observed improved fusion rates and reduced ODI scores after PISF, though without clear details on dosage or duration [37]. Waikakul et al. administered 600 IU/day of Vit.D2 for 10 days followed by 600 IU/day of Vit.D3 for maintenance in failed back surgery syndrome, noting symptomatic improvement despite the small sample size [33]. A meta-analysis reported no benefit for fractures, falls, or BMD, with no difference observed between doses above or below 800 IU/day [38]. These mixed findings align with broader orthopedic literature, where Vit.D3 supplementation shows limited or inconsistent effects on fracture healing and clinical outcomes, with positive results often linked to lower-quality studies or lacking dose–response clarity [6,7,8].

### 4.2. Global Variability and Clinical Uncertainty

International guidelines for Vit.D3 supplementation differ substantially in both dosage and age-group definitions. Some make no age distinctions, while others define multiple age brackets but recommend nearly identical adequate intake levels. Across all guidelines, the gap between the adequate intake and the upper level of intake (4000 IU/day) remains large. This complicates clinical decisions, particularly for spinal surgeons managing patients at risk of path-VF. For example, the United States recommends 600 IU/day for adults aged 19–70 years and 800 IU/day for those aged ≥71 years [15]. The European Food Safety Authority advises 600 IU/day for all adults and sets an upper level of intake of 4000 IU/day, while the German osteoporosis S3 guideline recommends 800 IU/day, advising against exceeding 2000–4000 IU/day [16,17]. The UK sets a range of 400–4000 IU/day for all adults without age subdivision [18]. South Korea recommends 400 IU/day for adults aged 19–49 years and 600 IU/day for those aged ≥50 years, with an upper limit of 4000 IU/day [19]. Japan recommends 360–4000 IU/day for all adults, subdivided into multiple age categories [39]. Such variability fosters subjective prescribing practices and underscores the lack of evidence-based dosing thresholds, a concern that is particularly relevant for spinal surgery patients.

### 4.3. Dose–Response Relationship in the Present Study

In this study, daily Vit.D3 intake showed a clear dose–response association with the risk of path-VF. Patients receiving supplementation at the lower end of guideline-recommended ranges had a substantially higher risk of path-VF. Consistent with this, patients in the path-VF group had significantly lower daily doses than those in the control group, with median values of 1431.4 IU versus 2366.7 IU, respectively (Table 2). This elevated risk for the Low-Dose group was evident in both the overall cohort and among patients above the identified age threshold of 67.5 years, with ORs indicating several-fold differences. The age distribution of supplementation in our cohort supports this interpretation because most patients in the path-VF group received around 1000 IU/day or less, particularly those above the threshold age, whereas higher-dose supplementation was more common among controls without path-VF. Based on this pattern, multiple logistic regression identified a threshold of approximately 1900 IU/day. One possible explanation is selection bias in supplementation practices; clinicians may preferentially initiate Vit.D3 at low doses without timely adjustment according to changes in the patient’s condition or individual needs.

The consistent dose–response trend indicates that low-dose regimens may be inadequate for high-risk patients. Doses above the threshold may help reduce vertebral fragility—related complications. However, as the identified threshold of approximately 1900 IU/day is based on retrospective data, prospective studies are needed to validate its clinical significance. Results from the High-Dose group should also be interpreted with caution, as the low rank-biserial correlation coefficients (*r* < 0.3) reflect limited statistical power due to small sample size. Additionally, the unexpectedly lower risk observed in the Zero-Dose group warrants careful interpretation and will be addressed separately.

### 4.4. Interpretation of Zero-Dose Patient Outcomes

The observed ORs below 1 in the Zero-Dose group should be interpreted cautiously and are unlikely to reflect a true protective effect of no supplementation. A more likely explanation is ‘healthy user’ bias, where these patients may have better bone quality, fewer comorbidities, or higher physical activity, even at older ages [40,41].

Structural changes associated with ageing, such as Modic changes, endplate sclerosis, and intervertebral osteophyte fusion, may also contribute to increased vertebral stiffness and improved screw stability. Wagnac et al. demonstrated that large osteophytes can enhance vertebral rigidity and reduce fracture susceptibility [42], while Modic-related sclerosis has been shown to increase HU values and prevent cage subsidence [43,44].

The benefit in the Zero-Dose group likely reflects selection bias and structural adaptations, though this remains speculative without objective health data.

### 4.5. Effect of Vit.D3 on HU Values of Vertebrae

At this stage of the analysis, we further explored whether the increased risk observed in the Low-Dose group could be explained by lower vertebral BMD assessed through HU measurements, and whether HU values differed systematically across the three dosage categories (Zero, Low, High). As expected, HU values were significantly lower in patients with path-VF than in controls, confirming their role as a surrogate marker of bone fragility and screw stability [45].

When analysing HU values across dosage categories, we observed that patients receiving Low-Dose Vit.D3 indeed showed lower HU values and were older than those without supplementation, whereas patients on High-Dose Vit.D3 displayed intermediate HU values without statistical significance. This finding is consistent with earlier clinical trials showing that low daily doses of Vit.D3, typically below 800–1000 IU, fail to improve BMD at the lumbar spine or hip [38,46], and that even moderate doses around 900 IU did not yield significant BMD increases in postmenopausal women [47,48].

These results suggest that while low-dose supplementation may be insufficient to counteract age-related BMD decline, higher doses do not necessarily translate into measurable HU gains either. The significant difference in Vit.D3 intake between path-VF and control groups (Table 2) therefore indicates that the observed clinical association is more closely related to overall dose adequacy. The apparent benefit of sufficient supplementation is likely to involve additional mechanisms beyond direct increases in vertebral BMD, such as modulation of bone metabolism, muscle function, or inflammatory pathways.

### 4.6. Potential Mechanisms of Vit.D in Preventing Path-VF Risk

The protective association of Vit.D3 observed in this study is unlikely to be mediated solely through increases in vertebral BMD. Instead, several additional biological mechanisms may contribute to the reduced path-VF risk. Beyond its established skeletal effects, Vit.D is involved in diverse physiological processes, including immune regulation, cardiovascular function, endocrine balance, and metabolic pathways. It has also been reported to alleviate pain, improve mood, and influence recovery after surgery [49].

One important pathway is through muscle function. Vit.D enhances muscle strength and balance, thereby lowering fall risk, a key determinant of fracture occurrence. This effect is mediated by Vit.D receptors in muscle cells, which regulate protein synthesis and calcium uptake [50]. Age-related declines in Vit.D receptor expression may precede structural bone changes, but Vit.D-related muscle weakness (hypovitaminosis D myopathy) can be reversed with adequate supplementation [51]. Clinical evidence supports this indirect protective effect, with studies showing that Vit.D supplementation reduces fall rates in older adults [52].

Vit.D also modulates parathyroid hormone activity, which is central to bone metabolism. Persistent Vit.D deficiency can lead to secondary hyperparathyroidism, causing elevated bone turnover and cortical bone loss. Pfeifer et al. demonstrated that Vit.D3 combined with calcium supplementation reduces parathyroid hormone levels in elderly women and improves balance, thereby decreasing fracture risk [53].

Finally, Vit.D exerts immunomodulatory and anti-inflammatory effects. It reduces circulating proinflammatory cytokines such as interleukin-6, interleukin-8, and tumour necrosis factor, which are known to impair bone homeostasis and hinder surgical recovery [54]. AlGhamdi et al. showed that high-dose monthly Vit.D3 administration (80,000 IU, approximately 2666 IU/day) significantly decreased these cytokines [55]. Such effects may not only contribute to fracture prevention but also enhance postoperative recovery and reduce complications such as cage subsidence or PSL after spinal procedures.

Taken together, these pleiotropic actions of Vit.D3 reinforce its clinical relevance in spinal surgery patients, particularly when direct improvements in vertebral bone density are not consistently measurable.

## 5. Limitations

This study has several limitations. First, its retrospective design limits the ability to establish causality between Vit.D3 dosage and path-VF occurrence. Second, serum 25(OH)D levels were not routinely assessed, preventing correlation analyses between measured Vit.D status and clinical outcomes. Third, the sample size in the High-Dose group was relatively small, which may have reduced the statistical power to detect significant associations.

Finally, other confounding factors, such as comorbidities, nutritional status, or physical activity levels, were not systematically controlled and may have influenced the observed relationships. In particular, the absence of objective health-status indicators introduces a potential ‘healthy user’ bias in the Zero-Dose group, meaning that their apparently lower risk may partly reflect better baseline physiological reserve rather than a true protective effect of no supplementation.

## 6. Conclusions

This study found a clear dose–response relationship between daily Vit.D3 intake and path-VF risk. Patients with path-VF received significantly lower Vit.D3 doses than controls. The elevated risk observed in the Low-Dose group, particularly in those over 67.5 years, suggests that commonly recommended minimum doses may not provide sufficient protection. A threshold of approximately 1900 IU/day may be relevant for risk reduction in elderly patients.

These effects are unlikely to result solely from improvements in BMD but may instead reflect broader physiological benefits of adequate Vit.D3 availability, including contributions to muscle strength, metabolic balance, and reduced inflammation. Overall, these findings highlight the potential value of tailored Vit.D3 supplementation strategies in spinal surgery patients at elevated risk of vertebral fragility. Further prospective studies with larger cohorts and systematic monitoring of serum 25(OH)D levels are needed to define and validate clinically relevant dosing thresholds.

## Figures and Tables

**Table 1:** Demographics of the involved patients.

		Age (Years)			
	*n*	Min.	Mean	Max.	SD
Total	210	18.5	67.5	87.9	15.4
Male	80	18.5	63.9	87.6	17.9
Female	130	23.1	69.7	87.9	13.1
Path-VF group	84	53.0	75.3	87.9	9.0
Male	22	53.0	74.0	87.0	10.6
Female	62	52.0	75.8	87.0	8.5
Control group	126	18.5	62.2	87.6	16.5
Male	58	18.5	60.0	87.0	18.7
Female	68	23.0	64.1	86.0	14.2
Zero Vit.D3-Suplement (0 IU/Day)	157	18.5	65.1	87.4	16.1
Low Vit.D3-Suplement (<2000 IU/Day)	33	53.0	75.4	87.7	9.2
High Vit.D3-Suplement (≥2000 IU/Day)	20	52.4	73.1	87.9	11.2
Diagnosed Osteoporosis	42	53.0	76.2	87.9	8.2
Osteoporosis with zero Vit.D3-Suplement	13	63.0	73.5	85.2	6.8
Osteoporosis with low Vit.D3-Suplement	20	53.0	77.7	86.8	9.1
Osteoporosis with high Vit.D3-Suplement	9	68.5	79.0	87.9	7.9
Patients with Hounsfield units Value	88	31.9	69.3	87.7	12.7

Path-VF: pathological vertebral fragility, SD: Standard deviation.

**Table 2:** Vit.D3 Doses in At-risk and Without-risk groups.

	*n*	Mean	SD	*p*	*r*
Path-VF group	35	1431.4	1055.7	<0.001 ***	0.36
Control group	18	2366.7	1186.7		

SD: standard deviation, *** *p* < 0.001.

**Table 3:** Multiple logistic regression.

	*β*	SE *β*	*z*	*p*	
(Intercept)	−4.34	2.94	−1.48	0.139	
Gender	0.64	0.92	0.69	0.490	
Vit.D3 Dose	−0.001	0.0004	−2.37	0.018	*
Age	0.08	0.04	2.15	0.031	*

SE: standard error, * *p* < 0.05.

## Data Availability

The datasets generated and/or analysed during the current study are not publicly available due to institutional data ownership. The data are the property of RKH Orthopaedic Clinic Markgröningen, 71706 Markgröningen, Baden-Württemberg, Germany. Data may be made available upon reasonable request and with prior approval from the institution. Requests should be directed to the corresponding author.

## Abbreviations

The following abbreviations are used in this manuscript:

25(OH)D	25-hydroxyvitamin-D
AUC	Area under the curve
BMD	Bone mineral density
CI	Confidence intervals
CT	Computed tomography
DEXA	Dual-energy X-ray absorptiometry
FF	Fragility fractures
HU	Hounsfield units
ODI	Oswestry disability index
OR	Odds Ratio
Path-VF	Pathological vertebral fragility
PISF	Posterior instrumented spinal fusion
PSL	Pedicle screw loosening
ROC	Receiver operating characteristic curve
SD	Standard deviation
SE	Standard error
VF	Vertebral fragility
VIF	Variance inflation factors
Vit.D	Vitamin D
Vit.D2	Vitamin D2
Vit.D3	Vitamin D3
